# Whole-Genome Epidemiology and Characterization of Methicillin-Susceptible *Staphylococcus aureus* ST398 From Retail Pork and Bulk Tank Milk in Shandong, China

**DOI:** 10.3389/fmicb.2021.764105

**Published:** 2021-11-30

**Authors:** Xiaonan Zhao, Ming Hu, Cui Zhao, Qing Zhang, Lulu Li, Yin Zhang, Yanbo Luo, Yuqing Liu

**Affiliations:** ^1^Institute of Animal Science and Veterinary Medicine, Shandong Academy of Agricultural Sciences, Shandong, China; ^2^Tai’an animal disease prevention and control center, Shandong, China

**Keywords:** methicillin-susceptible *Staphylococcus aureus*, sequence type 398, antimicrobial susceptibility testing, whole-genome epidemiology, *spa*

## Abstract

*Staphylococcus aureus* (*S. aureus*) is now regarded as a zoonotic agent. Methicillin-susceptible *S. aureus* (MSSA) ST398 is a livestock-associated bacterium that is most prevalent in China, but there are currently no data available for Shandong. Therefore, the aim of this study was to investigate the epidemiology and characterization of MSSA ST398 from retail pork and bulk tank milk (BTM) in Shandong. A total of 67 *S. aureus* isolates were collected from retail pork between November 2017 and June 2018. Among the isolates, high antimicrobial resistance rates were observed for penicillin (97.0%), and 92.5% of the isolates were multi-drug resistant (MDR). Eight sequence types (STs) were identified in the retail pork isolates, and the predominant type was ST15 (*n*=26), which was followed by ST398 (*n*=14). Staphylococcal protein A gene (*spa*) typing identified *spa* types t034 and t1255 in MSSA ST398 from retail pork. Using whole-genome sequencing analysis, we described the phylogeny of 29 MSSA ST398 isolates that were obtained from retail pork (*n*=14) and BTM (*n*=15). The phylogenetic tree showed that the MSSA ST398 isolates from different sources had the same lineage. Among the 29 MSSA ST398 isolates, five resistance genes were detected, and all isolates carried *DHA-1*. Fifteen toxin genes were detected, and all isolates carried *eta*, *hla*, and *hlb*. In conclusion, this study found that a high risk for MSSA ST398 was present in retail pork and BTM. These findings have major implications for how investigations of MSSA ST398 outbreaks should be conducted in the One-Health context.

## Introduction

*Staphylococcus aureus* is a well-known commensal pathogen of many animal species, including humans, that causes community and nosocomial infections (Li et al., 2018). It is regarded as one of the world’s leading causes of food consumption-related disease outbreaks (Abdalrahman et al., 2015). Previous studies have revealed that *S. aureus* has often been isolated from raw milk and retail meat (Johler et al., 2018; Sahibzada et al., 2018).

*S. aureus*-associated food poisoning in humans and similar intramammary infections in animals are produced by strains with the capacity to produce a wide range of virulence factors, including enterotoxins (*se*); toxic shock syndrome toxin-1 (*tst*); exfoliative toxins (*eta* and *etb*); leukocidins (*lukD/E/FS)*; and haemolysins (*hla*, *hld*, *hlg*, and *hlgv*; Le Marechal et al., 2011; Wilson et al., 2011). The role of virulence factors in disease causation is not as definitive as foodborne illness, and a plethora of factors are likely to be involved in disease pathogenesis (Fijalkowski et al., 2013).

Most *S. aureus* isolates are host-specific, and the potential for animals to act as a source of *S. aureus* infections for humans has been shown for some clonal lineages, such as ST398 (Mediavilla et al., 2012; Yan et al., 2014). Methicillin-resistant *S. aureus* (MRSA) ST398 can colonize animals and humans and can be transmitted between them (Li et al., 2015). MRSA ST398 has been isolated in Australia (Mitchell et al., 2014), New Zealand (Deborah et al., 2014), and Spain (Lozano et al., 2012). MSSA ST398 has been recovered from pigs in Germany and northeast China (Kadlec and Schwarz, 2010; Yan et al., 2014), humans in France and Taiwan, China (Rasigade et al., 2010; Huang and Chen, 2020), and retail meat in USA and Shanxi, China (Waters et al., 2011; Li et al., 2015). Recently, infections caused by MSSA ST398 have been described in humans (Valentin-Domelier et al., 2011; Mediavilla et al., 2012; David et al., 2013), and MSSA ST398 has been associated with efficient transmission between people, with greater capacity for adhesion to human skin (Uhlemann et al., 2012).

The food safety challenge we have faced during the current COVID-19 pandemic has increased concerns about the increasing numbers of outbreaks of staphylococcal food-borne intoxication. As a commensal bacterium in livestock, there has been little focus on MSSA ST398 contamination of retail meat and BTM in China. Therefore, the main objective of this study was to investigate the population structure of MSSA ST398 in retail pork and BTM by using whole-genome sequencing.

## Materials and Methods

### Bacterial Isolates From BTM

A total of 15 MSSA ST398 (SA-N1-N15) isolates from BTM in Shandong dairy farms were used in this study. Among the 15 MSSA ST398, 6 isolates were from Weifang, 3 isolates were from Jinan, 2 isolates were from Linyi, 2 isolates were from Dezhou, 1 isolate was from Rizhao, and 1 isolate was from Tai’an. Antimicrobial susceptibility testing and molecular typing were performed, as previously described (Zhao et al., 2021).

### Bacterial Isolation and Identification From Retail Pork Meat

From November 2017 to June 2018, a total of 200 retail pork samples were randomly purchased from two supermarkets (100 samples per supermarket) located in Tai’an and Jinan city. Each 25-g retail pork sample was aseptically placed into a sterile Whirl-Pak bag (Nasco, United States) and labelled. All collected samples were transported to the laboratory on ice within 6h after collection and were processed immediately. Isolation of *S. aureus* was performed as described by the following experimental procedure with some modifications (Sergelidis et al., 2012; Tang et al., 2017). For the isolation and detection of *S. aureus*, 25g of the retail pork samples were enriched in 100ml of trypticase soy broth (TSB, OXOID, United Kingdom) containing 6.5% NaCl and incubated at 37°C for 24h. Then, a loopful from the incubated tubes was streaked onto Baird-Parker Agar (Hopebiol, Qingdao, China) that was supplemented with 5% egg yolk and tellurite and incubated at 37°C for 24h. The suspected colonies with typical black appearances and surrounded by clear zones were identified as *S. aureus*. The suspected colonies were confirmed using the Stap identification system (bioMérieux, Marcy-l Étoile, France) and were further identified *via* amplification of the species-specific *nuc* gene using a previously described primer set (Louie et al., 2000).

### Antimicrobial Susceptibility Testing

All *S. aureus* isolates obtained from retail pork were subjected to antimicrobial susceptibility tests against 18 antimicrobial agents on Muller-Hinton agar with the agar dilution method by following the guidelines of the Clinical and Laboratory Standard Institute (Clinical and Laboratory Standards Institute, 2019). The antimicrobial agents used in this study were penicillin (PEN), amoxicillin/clavulanic acid (AMC), erythromycin (ERY), clindamycin (CLI), enrofloxacin (ENR), ofloxacin (OFL), ceftiofur (EFT), cefoxitin (FOX), sulfisoxazole (SF), vancomycin (VAN), trimethoprim/sulfamethoxazole (SXT), doxycycline (DOX), florfenicol (FFC), tiamulin (TIA), oxacillin (OXA), tilmicosin (TIL), gentamicin (GEN), and linezolid (LEZ). *S. aureus* ATCC25923 was used as the quality control strain. Antimicrobial resistance was defined as resistance to one or more classes of antimicrobials, whereas MDR was defined as resistance to three or more classes of antimicrobials.

### Molecular Typing

All of the isolates from retail pork were typed by multilocus sequence typing (MLST; Enright et al., 2000). The following seven housekeeping genes were used in the MLST scheme: carbamate kinase (*arcC*), shikimate dehydrogenase (*aroE*), glycerol kinase (*glp*), guanylate kinase (*gmk*), phosphate acetyltransferase (*pta*), triosephosphate isomerase (*tpi*), and acetyl coenzyme A acetyltransferase (*yqiL*). The amplicons were purified and sequenced (Invitrogen, Beijing, China). The alleles and STs were assigned according to the criteria of the MLST database.^1^ All typing data were imported into the BioNumerics software v6.5 (Applied Maths, Kortrijk, Belgium), clustered using the appropriate settings and the relationships displayed using graphing method called minimum spanning tree as described before (Schouls et al., 2005). All ST398 *S. aureus* isolates were investigated by *spa* typing (Harmsen et al., 2003). Typing was performed through the publicly available Ridom SpaServer.^2^

### Detection of Methicillin Resistance Genes

The genomic DNA of *S. aureus* isolates from the retail pork samples was extracted with a TIANamp Bacterial DNA extraction kit (Tiangen, Beijing, China). The presence of the *mecA* and *mecC* genes was tested by polymerase chain reactions (PCR) using primers and conditions as previously reported (Zdragas et al., 2015).

### Whole Genome Sequencing and Phylogenetic Analysis

All identified ST398 *S. aureus* isolates from retail pork and BTM were subjected to whole genome sequencing (Bankevich et al., 2012). Genomic DNA from each isolate was purified on DNeasy columns (Qiagen), and then sequenced on an Illumina MiSeq sequencer (Illumina, San Diego, CA, United States), with 100-base paired-end reads and barcodes within the Nextera XT DNA Library Preparation Kit (Illumina, United States). Read sequence quality was assessed with the Fastqc program^3^ and reads were quality-filtered with fastq-mcf (Ea-utils: https://expressionanalysis.github.io/ea-utils/). Genome assembly was performed with Edena v3 assembler (Hemandez et al., 2014). The assembled genomes were annotated with Prokka v1.10 software (Seemann, 2014). The Illumina sequence reads have been deposited in NCBI’s short read archive and the study accession number is shown in Supplementary Table S1. MUSCLE v3.8.31 software was used to compare the multiple sequences of the core genomes, and the results of these comparisons were used to construct the phylogenetic tree. The phylogenetic tree was constructed by using the maximum likelihood method with phyml v3.0.^4^ The ST398 isolates were investigated for all resistance and virulence genes in the *de novo* assembled contigs using ResFinder v2.1 (Zankari et al., 2012) and VirulenceFinder v1.5 (Joensen et al., 2014), respectively. Furthermore, the selected virulence genes (e.g., *sea ser*, *eta*, *etb*, *hla*, *hlb*, *hld*, *hlg*, *hlgv*, *tst*, *lukM*, *lukE*, *lukD*, *lukS*, and *lukF*) and resistance genes (e.g., *blaZ*, *DHA-1*, *msrA*, *ermA*, *ermB*, *ermC*, *ermT*, *tetK*, *tetL*, *tetM*, *vanA*, *fusB*, *far*, *dfrA*, *dfrG*, *aacA-aphD*, *aac (6′)-Ie-aph (2″)-Ia*, *mupA*, and *mupB*) were investigated by mapping the reference genes (Chairat et al., 2015; Ronco et al., 2018). The final phylogenetic tree and data of the carried resistance and virulence genes were input into the interactive Tree Of Life^5^ for further annotation (Xue et al., 2020).

## Results

### Bacterial Isolation and Identification

Among the 200 retail pork samples, a total of 67 *S. aureus* (67/200, 33.5%) were isolated, which included 42 isolates (42/100, 42.0%) from the Tai’an supermarket and 25 isolates (25/100, 25.0%) from the Jinan supermarket.

### Antimicrobial Susceptibility Testing

The susceptibility of the 67 *S. aureus* isolates to 18 antimicrobial agents was assessed (Table 1). Resistance to PEN (97.0%) was the most commonly observed in the retail pork isolates. High rates of resistance were also noted for ERY (86.6%) and FFC (83.4%). In contrast, all isolates were susceptible to AMC. The MDR phenotype was observed in 92.5% of the isolates. In addition, the MSSA ST398 isolates from BTM were susceptible to AMC, EFT, FOX, SXT, DOX, TIA, OXA, GEN, and LEZ. But most isolates were resistant to PEN (93.3%), OFL (93.3%), and ERY (86.7%, Table 1).

**Table 1:** Number and percentage of antimicrobial resistance of *S. aureus* isolated from retail pork and BTM.

Antimicrobial agents	Antimicrobial	No. of resistant isolates from retail pork (%)	No. of resistant isolates from BTM (%)
β-Lactams	Penicillin	65(97.0%)	14(93.3%)
	Amoxicillin / clavulanic acid	0	0
	Ceftiofur	7(10.4%)	0
	Cefoxitin	3(4.5%)	0
	Oxacillin	1(1.5%)	0
Macrolides	Erythromycin	58(86.6%)	13(86.7%)
	Tilmicosin	13(19.4%)	0
Lincomycin	Clindamycin	17(25.4%)	10(66.7%)
Quinolones	Enrofloxacin	19(28.4%)	9(60.0%)
	Ofloxacin	24(35.8%)	14(93.3%)
Sulfonamides	Sulfaisoxazole	44(65.7%)	3(20.0%)
	Trimethoprim/sulfamethoxazole	19(28.4%)	0
Glycopeptide	Vancomycin	9(13.4%)	1(6.7%)
Tetracyclines	Doxycycline	6(9.0%)	0
Chloramphenicol	Florfenicol	56(83.4%)	3(20.0%)
Diterpenes	Tiamulin	5(7.5%)	1(6.7%)
Aminoglycosides	Gentamicin	18(26.9%)	0
Oxazolidinones	Linezolid	9(13.4%)	0

### Molecular Typing

**Figure 1:**
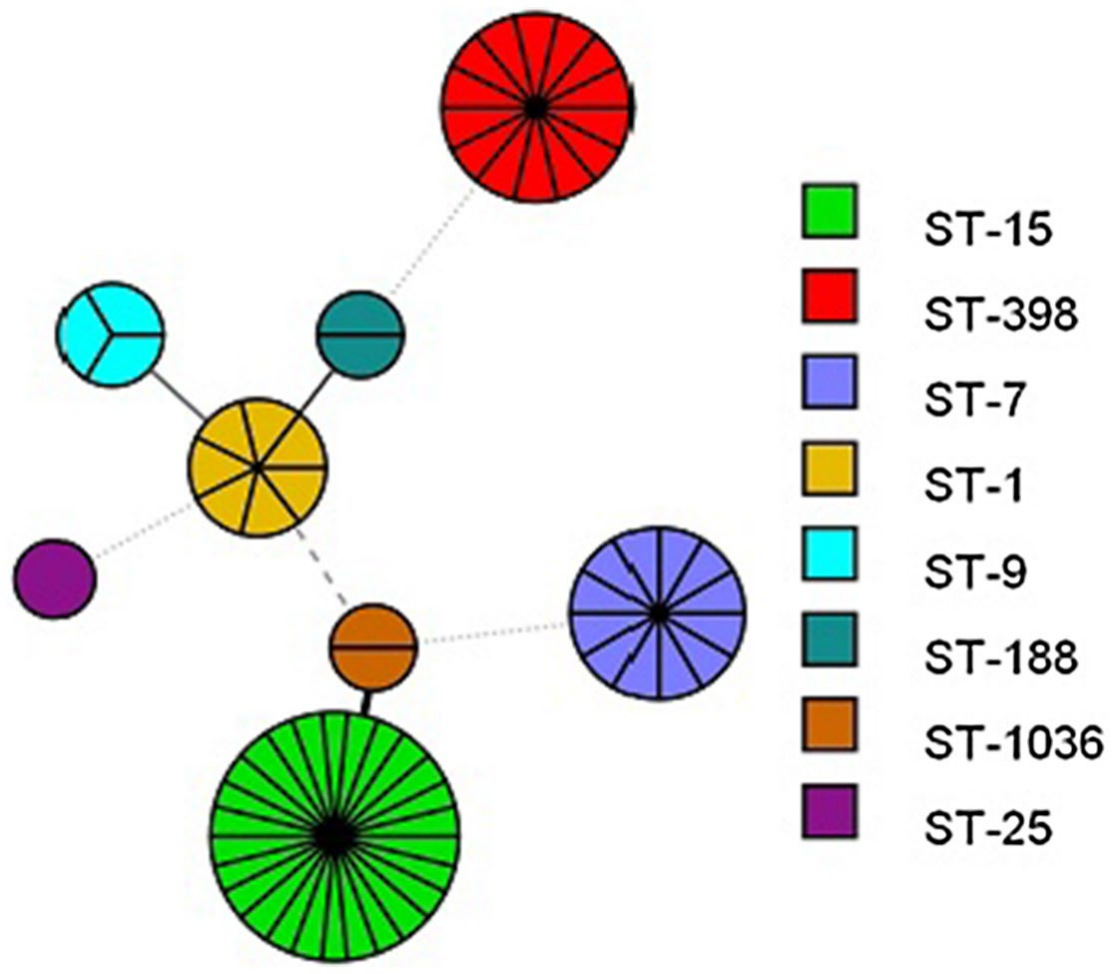
Minimum Spanning Tree based on the MLST data for each isolate. Numbers indicate ST of each node.

**Figure 2:**
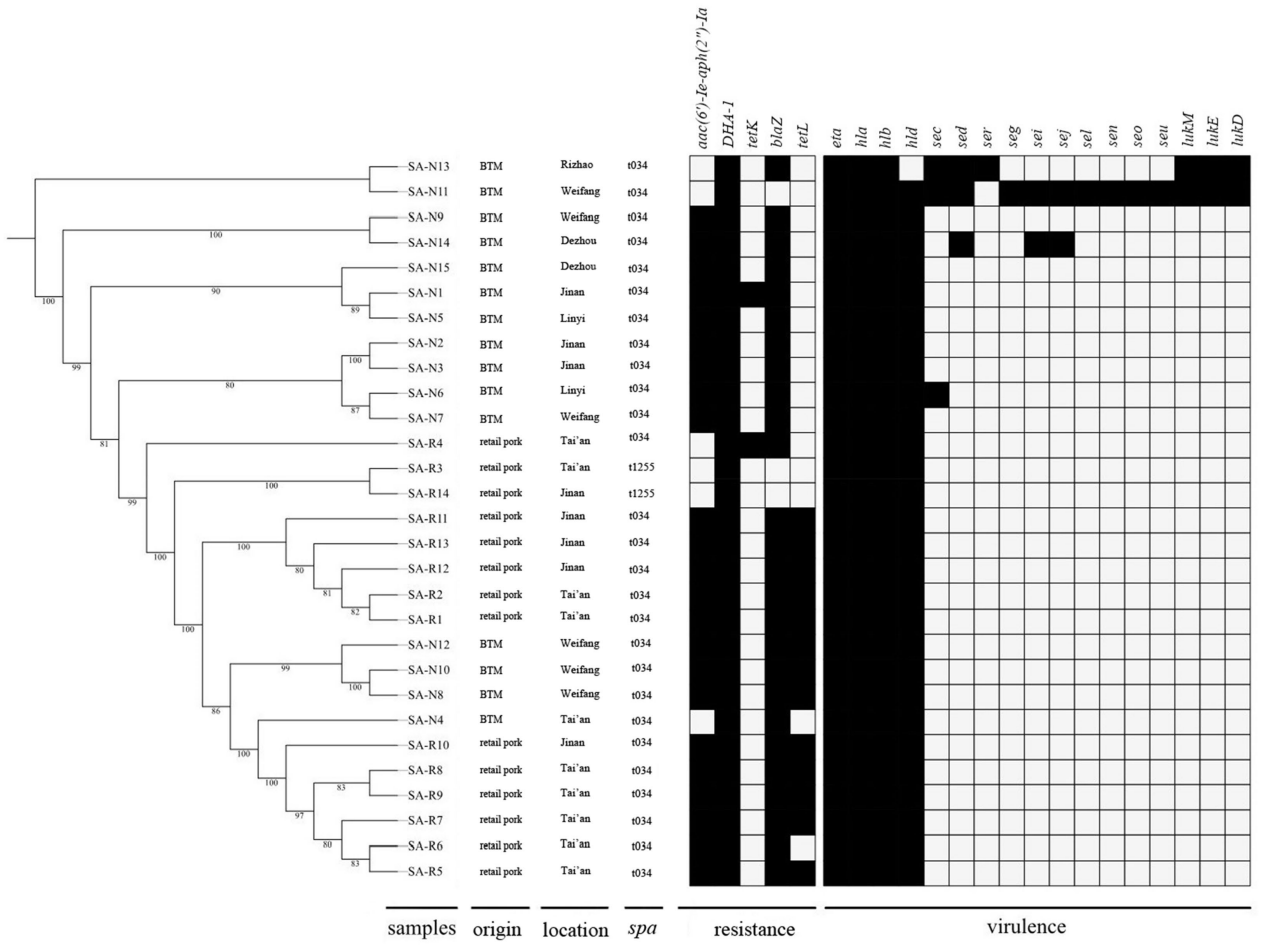
The distributions of the virulence and resistance genes identified in the 29 MSSA ST398 isolates from retail pork and BTM.

The MLST results revealed that a total of eight STs were identified in the retail pork isolates, including ST398, ST1036, ST25, ST15, ST7, ST1, ST9, and ST188 (Figure 1). Among them, ST15 was the most frequent genotype that was recovered from both supermarkets and involved 26 *S. aureus* isolates, which was followed by ST398 (*n*=14). The fourteen ST398 isolates from retail pork isolates were assigned to two different *spa* types. Most isolates belonged to t034 (*n*=12) and were followed by t1255 (*n*=2). In addition, the MSSA ST398 isolates from BTM all belonged to t034 (Figure 2).

### Detection of Methicillin Resistance Genes

None of the *S. aureus* isolates from the retail pork carried *mecA* and *mecC*, and they were all MSSA.

### Whole Genome Sequencing and Phylogenetic Analysis of MSSA ST398

The core genome-based phylogeny of the 29 MSSA ST398 isolates from retail pork (SA-R1-R14) and BTM is shown in Figure 2. In general, the phylogenetic tree showed that the MSSA ST398 isolates from different sources had the same lineage. In most cases, isolates from the same source clustered together. In general, the virulence and resistance genes were identified with thresholds of 90% nucleotide identity and 90% coverage of the query sequence length. The distributions of the virulence and resistance genes that were carried by these strains are shown in Figure 2. The resistance profiles were distinct between the MSSA ST398 isolates from retail pork and BTM, while the toxin profiles were relatively similar. Among the 29 MSSA ST398 isolates, five resistance genes were detected, and all strains carried *DHA-1*, which was followed by *blaZ* (*n*=26), *aac (6′)-Ie-aph (2″)-Ia* (*n*=23), *tetL* (*n*=13), and *tetK* (*n*=2). Fifteen toxin genes were detected, and all isolates carried *eta*, *hla*, and *hlb*, which were followed by *hld* (*n*=28), *sec* (*n*=3), *sed* (*n*=3), *sei* (*n*=2), *lukE* (*n*=2), and *lukD* (*n*=2), while *seg*, *sej*, *seo*, *seu*, and *ser* were represented only once.

## Discussion

In this study, we carried out whole-genome sequencing to investigate the phylogeny and characterization of MSSA ST398 from retail pork and BTM in China. To our knowledge, this is the first time that such type of MSSA ST398 isolates have been whole-genome sequenced and made publicly available. The prevalence of *S. aureus* in retail pork products from other studies has ranged from 12.0 to 59.7% (Hanson et al., 2011; Kelman et al., 2011; Waters et al., 2011). The results from this study were similar to those in other published reports, since *S. aureus* was found in 33.5% of retail pork, and they also coincide with other reports on poultry products that the prevalence of *S. aureus* in poultry products that has been reported worldwide has ranged from 23.5 to 50.5% (Ou et al., 2017). The isolates from the retail pork samples may originate from *S. aureus*-positive animals, the surrounding environment, humans, and other sources during processing and commercialization in meat counters at supermarkets and retail stores. Our results indicated that these meat items may serve as reservoirs of *S. aureus*, as with other retail meat samples.

Due to the excessive use of antibiotics, the prevalence of drug-resistant bacteria is increasing. In this study, we observed high resistance rates. The resistance rates to PEN (97.0%), ERY (86.6%), and FFC (83.4%) that were detected in this study have commonly been reported among *S. aureus* isolates from meat samples (Buyukcangaz et al., 2013; Ge et al., 2017). These high resistance rates may be related to the use of antimicrobials for treating disease and as growth promoters or feed additives in livestock. In this study, the MDR isolate rate of *S. aureus* (92.5%) from retail pork was consistent with the report that indicated that 90.0% of the strains were isolated from ready-to-eat meat sandwiches in Egypt (Mahmoud et al., 2021). However, this rate was higher than those reported for BTM (55.4%) and retail ready-to-eat foods (75.8%) in China (Zhao et al., 2021; Yang et al., 2016). Retail pork that is contaminated by MDR *S. aureus* is potentially hazardous, and the food chain may be the key site where resistance is transmitted between the environment and humans. In this regard, it was estimated that 2.8 million patients in the USA will be treated each year for resistant bacteria, and more than 35,000 will die as a result (CDC, 2019).

Several molecular typing methods were used to characterize the isolates, including MLST and *spa* typing. MLST is a DNA sequencing technology that uses sequence analyses of housekeeping genes to discriminate between isolates. MLST also offers the advantage that it is highly reproducible, which makes it an excellent tool for global comparisons of population structures (Enright et al., 2000). *spa* typing is specific to staphylococci and analyses the polymorphisms in the protein A gene (Frenay et al., 1996). In this study, eight STs were identified, and ST15 was the predominant type, which was different from the results of other studies. Previous studies have reported that ST5 has been found in retail pork in the United States, while ST9 was predominantly obtained from Asian countries, including China (Bhargava et al., 2011; Li et al., 2017). Importantly, it has been reported that MSSA ST398 isolates collected from retail pork are primarily associated with community- and hospital-acquired MSSA infections in humans (Chen and Huang, 2014). Two *spa* types were identified among the MSSA ST398 isolates from retail pork, and the predominant type was t034, which is a common and dominant type in Europe and North America (Pantosti, 2012).

The phylogenetic analysis showed that MSSA ST398 from retail pork clustered together with MSSA ST398 from BTM, which suggested that ST398 has a wide range of hosts and is considered to be adapted to the colonization of nonhuman mammals (Golding et al., 2010). In this study, we found that all MSSA ST398 isolates carried *DHA-1*, which belongs to class C of the Ambler classification and to group 1 of the functional classification of Bush, Jacoby, and Medeiros. These results were different from those of another study in which, among the 56 *S. aureus* isolates, only one in Iran carried *DHA-1* (Shahnaz et al., 2016). In our study, most of the MSSA ST398 isolates harboured *blaZ* (26/29), which was consistent with the result that most isolates were resistant to PEN. This observation is in line with a previous report that the *bla*Z gene confers PEN resistance (Johler et al., 2018). According to the results of this study, we suggested that MSSA ST398 could be a reservoir for the resistance genes. With regard to the risk of pathogenicity, the presence of virulence genes among the MSSA ST398 isolates was assessed in this study. All isolates carried *eta*, *hla*, and *hlb*, which coincided with the results of another study (Sun et al., 2019). SEs in particular are involved in human food poisoning (Hennekinne et al., 2012). In this study, we only detected SEs in BTM but none in retail pork, which can be attributed to the differences in geographical regions, sample sources and environments and needs to be further monitored. The detection of virulence genes in MSSA ST398 isolates reveals the lurking threat of retail pork and BTM, suggesting the need for implementing surveillance programs and prevention strategies.

## Conclusion

Our findings suggested that the retail pork examined was highly contaminated with *S. aureus*. In addition, the MSSA ST398 harboured multiple virulence and exhibited multiple antimicrobial resistance. To our knowledge, this is the first report of the detection of MSSA ST398 in retail pork and BTM in China. The whole genome sequencing studies are crucial to survey the global epidemiology of infectious agents, including MSSA ST398, and provided a deeper knowledge of the epidemiology of this bacterium and may help in understanding how to prevent and treat infections without boosting antibiotic resistance.

## Data Availability Statement

The datasets presented in this study can be found in online repositories. The names of the repository/repositories and accession number(s) can be found in the article/Supplementary Material.

## Ethics Statement

Ethical review and approval was not required for the animal study because The Ethical Statement is not applicable because sample collection from animals has been gathered.

## Author Contributions

XZ and YLi designed the work. XZ, MH, YZ, QZ, LL, and YLu collected samples. XZ and YLi analyzed and interpreted data. XZ drafted the article. XZ and YLi critically reviewed the article. XZ and CZ revised the manuscript. All authors contributed to the article and approved the submitted version.

## Funding

This work was supported by the National Key Research and Development Project (2019YFA0904004); Shandong Agricultural Major Applied Technology Innovation Program (SD2019XM009); Major Scientific and Technological Innovation Project in Shandong Province (2019JZZY010719); and The High-Level Talents and Innovative Team Recruitment Program of the Shandong Academy of Agricultural Sciences, China (CXGC2018E10).

## Conflict of Interest

The authors declare that the research was conducted in the absence of any commercial or financial relationships that could be construed as a potential conflict of interest.

## Publisher’s Note

All claims expressed in this article are solely those of the authors and do not necessarily represent those of their affiliated organizations, or those of the publisher, the editors and the reviewers. Any product that may be evaluated in this article, or claim that may be made by its manufacturer, is not guaranteed or endorsed by the publisher.

## Footnotes

^1^ http://saureus.mlst.net

^2^ www.spaserver.ridom.de

^3^ http://www.bioinformatics.babraham.ac.uk/projects/fastqc/

^4^ http://www.atgcmontpellier.fr/phyml/

^5^ http://itol.embl.de
