# Supplementary material for: Whole-Genome Epidemiology and Characterization of Methicillin-Susceptible Staphylococcus aureus ST398 From Retail Pork and Bulk Tank Milk in Shandong, China
Source: Front Microbiol. 2021 Nov 30;12:764105. doi: 10.3389/fmicb.2021.764105 (PMC8670001; doi:10.3389/fmicb.2021.764105)
Supplement: Supplementary file 1 [file Table_1.DOCX]

Table S1. Resistance genes, virulence genes and *spa* type in MSSA ST398 isolated from retail pork and BTM.

| No. | Origin | *spa* type | Resistance genes | Virulence genes | Accession numbers |  |  |  |  |  |  |  |
| --- | --- | --- | --- | --- | --- | --- | --- | --- | --- | --- | --- | --- |
| SA-N1 | BTM | t034 | *aac(6')-Ie-aph(2'')-Ia, DHA-1, tetK, blaZ* | *eta, hla, hlb, hld* | JAHVAQ000000000 |  |  |  |  |  |  |  |
| SA-N2 | BTM | t034 | *aac(6')-Ie-aph(2'')-Ia, DHA-1, tetK, blaZ* | *eta, hla, hlb, hld* | JAHVYF000000000 |  |  |  |  |  |  |  |
| SA-N3 | BTM | t034 | *aac(6')-Ie-aph(2'')-Ia, DHA-1, tetK, blaZ* | *eta, hla, hlb, hld* | JAHVYG000000000 |  |  |  |  |  |  |  |
| SA-N4 | BTM | t034 | *DHA-1, blaZ* | *eta, hla, hlb, hld* | JAHVYH000000000 |  |  |  |  |  |  |  |
| SA-N5 | BTM | t034 | *aac(6')-Ie-aph(2'')-Ia, DHA-1, blaZ* | *eta, hla, hlb, hld* | JAHVYI000000000 |  |  |  |  |  |  |  |
| SA-N6 | BTM | t034 | *aac(6')-Ie-aph(2'')-Ia, DHA-1, blaZ* | *eta, hla, hlb, hld, sec* | JAHVYJ000000000 |  |  |  |  |  |  |  |
| SA-N7 | BTM | t034 | *aac(6')-Ie-aph(2'')-Ia, DHA-1, blaZ* | *eta, hla, hlb, hld* | JAHVYK000000000 |  |  |  |  |  |  |  |
| SA-N8 | BTM | t034 | *aac(6')-Ie-aph(2'')-Ia, tet(L), DHA-1, blaZ* | *eta, hla, hlb, hld* | JAHVYL000000000 |  |  |  |  |  |  |  |
| SA-N9 | BTM | t034 | *aac(6')-Ie-aph(2'')-Ia, DHA-1, blaZ* | *eta, hla, hlb, hld* | JAHVYM000000000 |  |  |  |  |  |  |  |
| SA-N10 | BTM | t034 | *aac(6')-Ie-aph(2'')-Ia, tet(L), DHA-1, blaZ* | *eta, hla, hlb, hld* | JAHVYN000000000 |  |  |  |  |  |  |  |
| SA-N11 | BTM | t034 | *DHA-1* | *eta, hla, hlb, hld, sec, sed, seg, sei, sej, sel, sen, seo, seu, lukM, lukE, lukD* | JAHVYO000000000 |  |  |  |  |  |  |  |
| SA-N12 | BTM | t034 | *aac(6')-Ie-aph(2'')-Ia, tet(L), DHA-1, blaZ* | *eta, hla, hlb, hld* | JAHVYP000000000 |  |  |  |  |  |  |  |
| SA-N13 | BTM | t034 | *DHA-1, blaZ* | *eta, hla, hlb, sec, sed, ser, lukM, lukE, lukD* | JAHVYQ000000000 |  |  |  |  |  |  |  |
| SA-N14 | BTM | t034 | *aac(6')-Ie-aph(2'')-Ia, DHA-1, blaZ* | *eta, hla, hlb, hld, sed, sei, sej* | JAHVYR000000000 |  |  |  |  |  |  |  |
| SA-N15 | BTM | t034 | *DHA-1, aac(6')-Ie-aph(2'')-Ia, blaZ* | *eta, hla, hlb, hld* | JAHVYS000000000 |  |  |  |  |  |  |  |
| SA-R1 | Retail pork | t034 | *aac(6')-Ie-aph(2'')-Ia, tet(L), DHA-1,blaZ* | *eta, hla, hlb, hld* | JAHVYT000000000 |  |  |  |  |  |  |  |
| SA-R2 | Retail pork | t034 | *aac(6')-Ie-aph(2'')-Ia, tet(L), DHA-1, blaZ* | *eta, hla, hlb, hld* | JAHVYU000000000 |  |  |  |  |  |  |  |
| SA-R3 | Retail pork | t1255 | *DHA-1* | *eta, hla, hlb, hld* | JAHVYV000000000 |  |  |  |  |  |  |  |
| SA-R4 | Retail pork | t034 | *DHA-1, tet(K), blaZ* | *eta, hla, hlb, hld* | JAHVYW000000000 |  |  |  |  |  |  |  |
| SA-R5 | Retail pork | t034 | *aac(6')-Ie-aph(2'')-Ia, tet(L), DHA-1,blaZ* | *eta, hla, hlb, hld* | JAHVYX000000000 |  |  |  |  |  |  |  |
| SA-R6 | Retail pork | t034 | *aac(6')-Ie-aph(2'')-Ia, tet(L), DHA-1, blaZ* | *eta, hla, hlb, hld* | JAHVYJ000000000 |  |  |  |  |  |  |  |
| SA-R7 | Retail pork | t034 | *aac(6')-Ie-aph(2'')-Ia, tet(L), DHA-1, blaZ* | *eta, hla, hlb, hld* | JAHVYZ000000000 |  |  |  |  |  |  |  |
| SA-R8 | Retail pork | t034 | *aac(6')-Ie-aph(2'')-Ia, tet(L), DHA-1, blaZ* | *eta, hla, hlb, hld* | JAHVZA000000000 |  |  |  |  |  |  |  |
| SA-R9 | Retail pork | t034 | *aac(6')-Ie-aph(2'')-Ia, tet(L), DHA-1, blaZ* | *eta, hla, hlb, hld* | JAHVZB000000000 |  |  |  |  |  |  |  |
| SA-R10 | Retail pork | t034 | *aac(6')-Ie-aph(2'')-Ia, tet(L), DHA-1, blaZ* | *eta, hla, hlb, hld* | JAHVZC000000000 |  |  |  |  |  |  |  |
| SA-R11 | Retail pork | t034 | *aac(6')-Ie-aph(2'')-Ia, tet(L), DHA-1, blaZ* | *eta, hla, hlb, hld* | JAHVZD000000000 |  |  |  |  |  |  |  |
| SA-R12 | Retail pork | t034 | *aac(6')-Ie-aph(2'')-Ia, tet(L), DHA-1, blaZ* | *eta, hla, hlb, hld* | JAHVZE000000000 |  |  |  |  |  |  |  |
| SA-R13 | Retail pork | t034 | *DHA-1* | *eta, hla, hlb, hld* | JAHVZF000000000 |  |  |  |  |  |  |  |
| SA-R14 | Retail pork | t1255 | *aac(6')-Ie-aph(2'')-Ia, tet(L), DHA-1, blaZ* | *eta, hla, hlb, hld* | JAHVZG000000000 |  |  |  |  |  |  |  |
